# Type II diabetes and personality; a study to explore other psychosomatic aspects of diabetes

**DOI:** 10.1186/s40200-016-0281-3

**Published:** 2016-12-03

**Authors:** Maryam Esmaeilinasab, Mehdi Ebrahimi, Mohsen Heidari Mokarrar, Leila Rahmati, Mohammad Yoosef Mahjouri, Seyed Masoud Arzaghi

**Affiliations:** 1 Department of Psychology, Tarbiat Modares University, Tehran, Iran; 2 Endocrinology and Metabolism Research Center, Endocrinology and Metabolism Research Institute, Tehran University of Medical Sciences, Tehran, Iran; 3 Department of Psychiatry, Zabol University of Medical Sciences, Zabol, Iran; 4 Fellowship in Psychosomatic Medicine, Elderly Health Research Center, Endocrinology and Metabolism Population Sciences Institute, Tehran University of Medical Sciences, Tehran, Iran; 5 EMRI, Dr Shariati Hospital, North Karegar St, Tehran, 14114 Iran

**Keywords:** Personality traits, Defense styles, Spirituality, Glycemic control, Type II diabetes

## Abstract

**Background:**

As one of the most common chronic diseases, diabetes and its control are affected by the patients’ psychological and spiritual attributes. The present study investigates the relationship between glycemic control in patients with type II diabetes and personality traits, defense mechanisms and spirituality.

**Method:**

The present cross-sectional study was conducted on 400 Iranian patients with type II diabetes, 64% were men. Participants completed the NEO Personality Inventory, the Defense Style Questionnaire (DSQ) and the Spiritual Assessment Inventory (SAI) and then underwent a blood sampling for the assessment of HbA1C levels.

**Results:**

Of the five personality traits, extraversion (*r =* -0.13 and *P <* 0.01) and conscientiousness (*r =* -0.13 and *P <* 0.01) had significant negative relationships with HbA1C HbA1C levels, while neuroticism had a significant positive relationship with HbA1C levels (*r =* 0.12 and *P <* 0.05). Of the defense styles assessed, the neurotic style was found to have a significant negative relationship with HbA1C levels (*r =* -0.1 and *P <* 0.05). Also, of the spirituality elements, impression management had significant relationship with glycemic control (*r =* 0.17 and *P <* 0.001).

**Conclusion:**

According to data, Extraversion and conscientiousness can help control blood sugar while anxiety and negative emotions have detrimental effects on glycemic control. As a result considering psychological counselling beside medical interventions can help to better treatment.

## Background

Diabetes is one of the most common chronic diseases that affect every aspect of a patient’s life. In recent years, lifestyle changes and the growing prevalence of obesity appear to have led to an increased prevalence of diabetes [1]. As a lifestyle-dependent variety, type II diabetes accounts for 90–95% of all types of diabetes [2]. The treatment of diabetes is focused on the control of glycosylated hemoglobin (HbA1C) and keeping it at normal levels as a major indicator of diabetes control [3].

Given the chronic nature of diabetes, achieving treatment goals for the disease depends mostly on the patient’s compliance with medical instructions and self-care activities; however, the patient’s compliance is affected by several factors as well. George Engel’s biopsychosocial model of chronic diseases and his argument that these diseases have to be understood more comprehensively have brought the role that psychological factors play in health and illness into attention [4]. The present study attempts to address the major psychological dimensions of human-beings that affect glycemic control. Type I and II diabetes are different in many aspects. Type I is more primary and as a result biological factors seem to have more crucial role in trends of diseases. Type II diabetes, on the other hand, mostly accurse in later ages and t seems life style and consequently psychological factors play important role in that beside biological potentiality. Considering this issue, we focused in this study on type II patients.

Given the role of psychological factors, the diabetic personality was first proposed in 1930. This idea assumes that diabetic personality traits transform and exacerbate the disease and complicate it by predisposing the patient to turn psychological tensions into physical reactions [5]. Lane et al. conducted a study on 105 patients with type II diabetes and found glycemic control to have a direct negative relationship with neuroticism and a direct positive relationship with the altruism of an extravert personality [6]. In a study conducted by Wheeler et al. on 28 patients with type I diabetes, insulin acceptance was reported to have a direct negative relationship with neuroticism and a direct positive one with conscientiousness [7]. In addition to personality traits, some studies have referred to defense mechanisms as a subconscious psychological tool for protection against life stressors that can have significant effects on glycemic control. These mechanisms are psychological measures for protection against stressful and threatening factors and affect the individual’s physical and psychological health [6–8]. Although defense mechanisms protect patients from the anxiety of their disease, they also diminish their perception of having a serious need for medical care [9]. Spirituality is another dimension of health that has recently come to be emphasized by the World Health Organization. Some studies have confirmed the existence of a relationship between spirituality and physical health [10–12]. Spiritual beliefs include the perception of a higher power and a sense of being connected to this source of power [11]. Some findings suggest that spiritually-inclined individuals adopt healthier methods of coping with psychological pressures, which in turn has a positive effect on their health. Individuals who score higher on spirituality are more likely to evaluate a stressful situation as positive [13]. In a qualitative study on diabetic patients, Cattic and Kudson demonstrated that adapting to diabetes depends on the meaning the individual attaches to the disease and his spiritual relationship with the Creator and also showed that spirituality determines people’s optimistic view of the disease and their efforts to manage it [12]. Rappaport argued that individuals who can find a positive spiritual meaning for their disease can better adapt to diabetes [14]. The main limitation of studies on spirituality concerns the non-generalizability of their results to other societies and cultures, since spirituality is a culture-dependent construct that should be investigated separately in each culture. It is therefore essential for Iranian studies on spirituality to be designed according to the dominant culture of Iran. The present study was conducted in response to the need for a multidimensional perspective on medical issues and the lack of studies on the psychological factors involved in diabetes. Although some studies have dealt with the subject, only a limited number have specifically addressed the relationship between personality traits, defense mechanisms and spirituality and the development and treatment of diabetes. This study thus seeks to examine glycemic control in patients with type II diabetes and its relationship with personality traits, defense mechanisms and spirituality and to identify their roles in diabetes treatment follow-up and to respond to the question, “Is there a significant relationship between glycemic control in patients with diabetes and personality traits, defense mechanisms and spirituality?”

## Methods

The present cross-sectional study was conducted on a study population of 18–65 year-old men and women with type II diabetes (without chronic physical diseases unrelated to diabetes or major psychiatric disorders such as schizophrenia, etc.) selected through convenience sampling and who had been under treatment for at least a year at the Endocrinology and Metabolism Research Institute of Dr. Shariati Hospital in Tehran. First, to assess their psychological indicators, eligible patients were referred to a psychologist and a psychiatrist by an endocrinologist to be checked for special psychological problem. Interview based on DSM criteria was the source for assessing. After the final confirmation of their eligibility for participation in the study, the researcher briefed participants on the study objectives and methods with the help of the fellow psychologist and psychiatrist. HbA1c measurement was performed via D-10 HbA1c (Bio-Rad Laboratories, Hercules, CA), Ion exchange HPLC method. Participants then submitted their informed written consents and completed the study questionnaires. The data collected were ultimately analyzed in SPSS-20.

### Data collection tools

Participants were assessed using the five-factor NEO Personality Inventory, the Defense Style Questionnaire (DSQ), the Spiritual Assessment Inventory (SAI) and a blood test:

1. *The five-factor NEO Personality Inventory – short form:* The revised short form of the NEO contains 60 items measuring the five dimensions of personality, including neuroticism (emotional stability), extraversion (introversion), openness to experience (secretiveness), agreeableness (argumentativeness) and conscientiousness (irresponsibility), which are scored based on a Likert scale. McGray & Costa reported the compatibility of the short and long forms with a correlation above 68%, also Egan et al. reported different Cronbach’s alpha values for the inventory, ranging from 72% for openness to experience to 87% for neuroticism and the validity and reliability of the Persian version of the inventory have also been confirmed [15].
2. *The Defense Style Questionnaire (DSQ):* This questionnaire contains 40 items and assesses 20 defense mechanisms at three levels, including mature, neurotic and immature levels. The long form of this questionnaire was developed in 1993 by Andrews et al. The psychometric features of the Persian version of the questionnaire indicate its favorable validity and reliability with Cronbach’s alpha values of 0.81 and 0.87 [16].
3. *The Spiritual Assessment Inventory (SAI):* This questionnaire was developed in 1996 by Hall & Edwards and contains the sub-scales of awareness, realistic acceptance, disappointment, grandiosity, instability and impression management, which have Cronbach’s alpha values of 0.95, 0.9, 0.83, 0.73, 0.8, and 0.77, in respective order. According to the Spiritual Well-Being and Coping Styles test, the concurrent validity of the inventory is favorable [17]. The results of the factor analysis carried out in the present study showed a reliability of 0.962 for the 45 items and no items were therefore eliminated. Cronbach’s alpha values obtained were 0.924 for the whole inventory and 0.88, 0.83, 0.82, 0.82, 0.71 and 0.71 for the sub-scales of awareness, realistic acceptance, disappointment, grandiosity, instability and impression management.

### Statistical analysis

The descriptive indicators of participants’ personality traits and test results were reported using the mean and standard deviation. The Pearson Correlation Coefficient was used to assess the relationship between participants’ HbA1C levels and their scores on the different personality traits, defense mechanisms and spirituality. SPSS-20 used for analyzing data.

## Results

Of the total of 400 participants, 256 (64%) were men and 144 (36%) were women. The mean age of the subjects was 51 (51.2 ± 8.8), 83.3% were married and 40% had only a high school diploma. The majority of participants (71.8% and *n =* 287) used glycemic control pills for their treatment and the rest had insulin injections along with these pills in their medical regimens (28.3% and *n =* 113). The duration of the disease varied from one year to 30 years and had a mean of 10.1 ± 6.6 years in participants. The mean HbA1C level as an indicator of glycemic control was 7 ± 1.7%, ranging from a minimum of 6.2% to a maximum of 10.6%, indicating a close to optimal control in participants. Examining the correlation between the different defense styles and the blood test indicators showed a significant negative correlation between neuroticism and HbA1C levels (*r =* -0.1 and *P <* 0.05). Examining the correlation between the different personality traits and the blood test indicators showed a significant positive correlation with neuroticism (*r =* 0.12 and *P <* 0.05) and significant negative correlations with extraversion (*r =* -0.13 and *P <* 0.01) and conscientiousness (*r =* -0.13 and *P <* 0.01). Examining the correlation between spirituality and the blood test indicators showed a significant positive correlation only with impression management (*r =* 0.17 and *P <* 0.001) (Table 1).

**Table 1:** Correlation between HbA1C levels and personality traits, defense styles and spirituality (*n =* 400)

Variable		HbA1C Level	*P-*Value
Personality Traits	Neuroticism	0.123*	0.02
	Extraversion	−0.125**	0.01
	Openness to Experience	−0.046	0.3
	Agreeableness	0.016	0.747
	Conscientiousness	−0.133**	0.008
Defense Styles	Neurotic	−0.1*	0.04
	Immature	−0.004	0.9
	Mature	−0.052	0.3
Spirituality	Awareness	0.0037	0.4
	Realistic Acceptance	−0.012	0.8
	Disappointment	−0.021	0.6
	Grandiosity	−0.055	0.2
	Instability	0.65	0.1
	Impression Management	0.169**	0.001

* *P <* 0.05

** *P <* 0.01

## Discussion

The psychological aspect of diabetes is considered an important part of the treatment and control of this condition in the modern world. Psychologically-neglected diabetics tend to not follow up adequately with the routine medical therapies for diabetes either. The present cross-sectional study was conducted to determine the relationship between glycemic control in patients with type II diabetes and personality traits, defense styles and spirituality. The results obtained revealed significant relationships between some of the more routine personality traits and glycemic control in diabetic patients.

A negative relationship was found between extraversion as a personality trait and HbA1C levels; that is, the more extravert was a patient, the lower were his HbA1C levels or the better was his glycemic control. This relationship might be explained by the six dimensions that exist to this personality trait, including warmth in relationships, sociability, assertiveness, activeness, excitement seeking and positive emotions. The positive attitude inherent to this personality trait can explain the better acceptance of diabetes and adaptation to it through self-care behaviors. The tendency toward sociability encourages patients with this personality trait to establish relationships with other diabetics and to better accept their disease. Moreover, sociability strengthens the desire to be part of social activities like sports settings, which helps with a better glycemic control. The findings of the present study are consistent with the results obtained by Wheeler et al. about patients with type I diabetes, which showed a significant positive relationship between extraversion and physical activity and exercise (an indicator of adherence to treatment) [7]. A significant negative relationship was found between conscientiousness and HbA1C levels; that is, higher scores in this parameter were associated with a better glycemic control. The six dimensions of conscientiousness include having competence and being systematic, responsible, hardworking, orderly, thorough, accurate and vigilant. Glycemic control and personal diabetes management require stringent adherence to one’s medical regimen and compliance with the doctor’s advice. Individuals with a conscientiousness personality trait have sufficient motivation for a proper glycemic control due to all the attributes they possess; moreover, their self-efficacy with respect to these attributes helps with their efforts to achieve their health goals. Being orderly and vigilant improves their motivation for accepting their treatment. The results obtained with respect to this personality trait are consistent with the results obtained by Brickman et al. [18], Booth-Kewley and Vickers [19] and Christensen and Smith [20]. Wheeler et al. found a significant relationship between scoring high on this personality trait and having a better insulin acceptance and diet adherence. Insulin acceptance was particularly dictated by self-efficacy and being orderly and vigilant, while diet adherence was particularly dictated by being orderly and vigilant [7]. The study also showed a significant relationship between the neuroticism personality trait and HbA1C levels. The dimensions of this personality trait include anxiety, anger and aggression, depression, self-consciousness, impulsivity and vulnerability; the majority of these attributes are accompanied with negative emotions. Depression, anxiety and anger can increase blood sugar levels physiologically; at the same time, it can have adverse effects on self-care behaviors in patients and thus diminish their treatment motivations and adherence to the medical regimen. Impulsivity leads to vulnerability in these individuals and reduces their tolerance of difficult situations, especially complicated diabetes treatment regimens, and increases their risk of treatment termination following any problems that may arise. These results are consistent with the results obtained by Brickman et al. [18]. Wheeler et al*.* also found a significant relationship between the score obtained for this personality trait and insulin acceptance, as patients who scored higher on this trait had a poorer insulin acceptance. This relationship was much more striking with respect to depression and anger [7]. Wheeler et al. also found a significant relationship between impulsivity and diet adherence, which was fairly expected [7].

The results of the present study also showed a significant relationship between neurotic defense styles and glycemic control. These defense styles are inflexible and tend to preoccupy the patient with negative emotions rather than directing him toward solving the problem. These defense styles tend to have adverse effects on self-care in patients with diabetes and can predispose them to non-adherence to their treatment regimen. The results obtained also showed that, Impression management subscales of spirituality had a significant positive relationship with glycemic control, which was not what we expected. One explanation for this relationship may be that personality traits and defense mechanism are stronger predictors of glycemic control in these patients, which is it considerable itself. Nevertheless, this relationship has to be further examined through other scales, for instance.

Limitations of the present study include the selection of participants from only two public clinics affiliated to Tehran University of Medical Sciences, and given the demographic(very wide range of age) and socioeconomic characteristics of participants, the results may not be easily extended to all diabetics; also this is a cross sectional study by which we can’t assess any aspect of causality and the sample size is small to capture the range of personality types; so future studies are therefore recommended to be population-based and to be conducted on a more public level with greater sample size. At last, it is necessary to mentioned that anyway chronic diseases can have itself effects on personality traits to some extents that I studies it should be considered. Also other aspects of diabetes control such as self-care behaviors (self-monitoring of blood glucose, diabetes medication compliance, smoking status, etc.) can be important as dependent variables which we suggest to consider in further studies.

## Conclusions

The results of the present study showed that investigating personality traits can be useful for identifying patients predisposed to improper glycemic control. These results can also be used in the design of educational programs for newly diagnosed diabetic patients. The assessment of personality traits can have a substantial role in the proper treatment of diabetics. Although personality traits are generally considered innate constructs that are resistant to change, more effective steps should be focused on making patients aware of their personality traits and its effects on good or bad trends of their illness and try to modify them through constant psychological interventions beside medical therapies. It should be noted that this study was conducted on patients at a wide age range and with a mean age of 51 years. As some studies argue that correlations change for different age-based factors, future studies are also recommended to account for the variable of age and to examine factors affecting treatment follow-up with respect to this variable.
